# Signal Construction-Based Dispersion Compensation of Lamb Waves Considering Signal Waveform and Amplitude Spectrum Preservation

**DOI:** 10.3390/ma10010004

**Published:** 2016-12-23

**Authors:** Jian Cai, Shenfang Yuan, Tongguang Wang

**Affiliations:** 1 Research Center of Structural Health Monitoring and Prognosis, State Key Lab of Mechanics and Control of Mechanical Structures, Nanjing University of Aeronautics and Astronautics, Nanjing 210016, China; caijian@nuaa.edu.cn; 2 Jiangsu Key Laboratory of Hi-Tech Research for Wind Turbine Design, Nanjing University of Aeronautics and Astronautics, Nanjing 210016, China; tgwang@nuaa.edu.cn

**Keywords:** structural health monitoring, Lamb waves, dispersion compensation, signal construction, damage imaging

## Abstract

The results of Lamb wave identification for the aerospace structures could be easily affected by the nonlinear-dispersion characteristics. In this paper, dispersion compensation of Lamb waves is of particular concern. Compared with the similar research works on the traditional signal domain transform methods, this study is based on signal construction from the viewpoint of nonlinear wavenumber linearization. Two compensation methods of linearly-dispersive signal construction (LDSC) and non-dispersive signal construction (NDSC) are proposed. Furthermore, to improve the compensation effect, the influence of the signal construction process on the other crucial signal properties, including the signal waveform and amplitude spectrum, is considered during the investigation. The linear-dispersion and non-dispersion effects are firstly analyzed. Then, after the basic signal construction principle is explored, the numerical realization of LDSC and NDSC is discussed, in which the signal waveform and amplitude spectrum preservation is especially regarded. Subsequently, associated with the delay-and-sum algorithm, LDSC or NDSC is employed for high spatial resolution damage imaging, so that the adjacent multi-damage or quantitative imaging capacity of Lamb waves can be strengthened. To verify the proposed signal construction and damage imaging methods, the experimental and numerical validation is finally arranged on the aluminum plates.

## 1. Introduction

To determine the integrity and reduce the life-cycle costs of critical aerospace structures, the concept of structural health monitoring (SHM) is increasingly acknowledged. SHM can permit the real-time and in-situ damage identification via distributed network sensors permanently mounted on or embedded into the structures [1,2,3,4,5]. As a kind of guided ultrasonic waves in thin-wall structures, Lamb waves can travel over large distances with high sensitivity to both the surface and internal defects. The suitability of Lamb waves for SHM of plate-like aerospace structures has been well demonstrated. Besides the classic ellipse or triangulation damage location, Lamb wave imaging of phased array [6,7], reverse-time migration [8], tomography [9], time reversal [10,11,12] or delay-and-sum [13,14], is frequently proposed and performed for metallic or composite structures. While in practical applications, the multi-mode and dispersion characteristics are the two most important issues needing to be properly addressed. Usually, a windowed toneburst with finite time duration is selected to generate single fundamental symmetric mode (S_0_) or anti-symmetric mode (A_0_) by tuning excitation frequency at a proper operating point on dispersion curves [15,16]. However, over the frequency range of such a narrowband excitation signal, neither the group nor phase velocities can maintain frequency-independence even at the operating point of “zero dispersion” [15]. The dispersion effect remains non-negligible. The Lamb wavepackets will spread out in time and space with their envelopes misshaped as they propagate [17]. This can explicitly affect the accuracy and resolution of Lamb wave monitoring.

Time reversal process (TRP), based on the principle of spatial reciprocity and time reversal invariance of linear wave equations, can automatically compensate the dispersion effect on Lamb waves [18,19]. Unfortunately, the traveling time of the compensated waves is also eliminated at the same time. This makes TRP less practical in damage detection because time-of-arrival (TOA) information is eliminated. To overcome this problem, virtual time reversal (VTR) [20] was introduced to partially recompress the dispersive wavepackets with time information retained. Using the priori-knowledge of dispersion characteristics of Lamb waves in the tested structures, a number of signal processing approaches have been developed for dispersion compensation and regularly applied in Lamb wave imaging.

Sicard et al. [21,22] proposed a numerical reconstruction method for time compaction of S_0_ mode signals in steel plates and applied the method to strengthen the small corrosion-pitting detectability of Lamb-synthetic aperture focusing technique (L-SAFT). Based on the assumption that dispersed waveforms get converged at t = 0 and then diverge again in the back-propagation direction, Wilcox solved the dispersion problem by mapping Lamb wave signals from the time to distance domains [23]. The time-distance domain mapping (TDDM) results subjected to inaccuracies of the supplied dispersion data were also analyzed. To perform TDDM in compact omni-directional array imaging [7,24], the single mode signals before or after phase addition were converted to the wavenumber domain. Hall [25] and Engholm [26] employed TDDM to advance the imaging capacity for adaptive beamforming of minimum variance distortionless response (MVDR). Pradoa et al. [27] utilized TDDM in Lamb mode diversity imaging for correct defect localization. Liu and Yuan [28] proposed the linear mapping (LM) technique to effectively recompress the dispersive A_0_ mode signals in aluminum plates. Xu et al. [29] compared TDDM and LM approaches. The suitability of TDDM for the embedded ultrasonic structural radar (EUSR) technique was also explored. Recently, a modified time-distance domain transform (TDDT) method is introduced and applied for dual damage imaging on an aluminum plate [30]. Marchi et al. [31] actualized dispersion compensation of Lamb waves with warped frequency transform (WFT) and performed the compensation procedure for impact location.

Except for LM, most of the above approaches are the signal domain transform ones, in which the former time-frequency domain dispersive signals are commonly transferred to the distance-wavenumber domain for dispersion compensation [21,23,30,31]. The signal domain transform methods, originally performed by Booer et al. [32] for the dispersed seismic waves guided by coal seams, have been widely used as the classical dispersion compensation methods for Lamb waves. However, in the methods, the influence of the compensation process on the other important signal properties, especially the signal waveform or the amplitude spectrum, closely related to the signal amplitude and physical damage sensitivity is usually neglected for simplicity. Then, the waveform deformation or the amplitude spectrum alteration can be easily brought about to the compensated signals. In TDDT [30] for instance, though the waveform correction is considered, the amplitude spectrum is found to be grievously altered to result in the severe deterioration of the signal SNR and damage sensitivity. This would probably create much inconvenience in the interpretation of the processed signals and decrease the compensation effect.

Considering the above problems and providing alternative efficient dispersion compensation approaches for high spatial resolution Lamb wave imaging of aerospace structures, the two compensation methods of linearly-dispersive signal construction (LDSC) and non-dispersive signal construction (NDSC) are comparatively presented in this paper, based on signal construction with the idea of nonlinear wavenumber linearization. During the investigation of LDSC and NDSC, the signal waveform and amplitude spectrum maintenance are particularly regarded to enhance the dispersion compensation effect. The remaining content is organized as follows: Section 2 theoretically and numerically analyzes the different dispersion effects on Lamb wave signals. In Section 3, LDSC and NDSC are proposed. Both the basic signal construction principle and numerical realization of LDSC and NDSC are discussed. Additionally, the two signal construction methods are compared with the typical dispersion compensation methods, i.e., TDDM and TDDT. In Section 4, LDSC or NDSC-based high spatial resolution imaging is developed. Section 5 conducts the experimental validation and numerical simulation to testify the proposed dispersion compensation and high spatial resolution imaging methods based on LDSC or NDSC. Conclusions are made in the last section.

## 2. Effects of Different Dispersion Relations on Lamb Waves

### 2.1. Sensing Model in Frequency Domain

To facilitate the theoretical investigation, a sensing model is firstly simplified in the frequency domain. With piezoelectric (PZT) wafers applied as actuators and sensors, a Lamb wave signal, supposed of single wavepacket for simplicity, can be represented in the frequency domain as [19,20]

(1) $$V_{0}(\omega )=V_{a}(\omega )H(\omega )$$

where ω, r, $V_{a}(\omega )$ and $V_{0}(\omega )$ are the angular frequency, propagation distance, frequency-domain excitation signal and sensor signal, respectively. H(ω), regarded as the transfer function of the whole procedure including Lamb wave exciting, propagating and sensing, can be expressed by

(2) $$H(\omega )=A(r,\omega )e^{-iK_{0}(\omega )r}$$

where A(r,ω) is actually the amplitude spectrum of H(ω), $K_{0}(\omega )$ is the wavenumber that determines the dispersion relation of the Lamb wave mode, and

(3) $$c_{p}(\omega )=\omega /K_{0}(\omega ),\;c_{g}(\omega )=d\omega /d\left[K_{0}(\omega )\right]$$

where $c_{p}(\omega )$ and $c_{g}(\omega )$ are the phase and group velocities of the mode, respectively.

With A(r,ω) simplified as “1” to ease the following analysis, the sensing model is derived as

(4) $$V_{0}(\omega )=V_{a}(\omega )e^{-iK_{0}(\omega )r}$$

In Equation (4), since $K_{0}(\omega )$ is usually nonlinear with ω, i.e., the Lamb wave mode is nonlinearly-dispersive. Different frequency components of $V_{0}(\omega )$ will have inconsistent time delays $K_{0}(\omega )r/\omega$ to make the wavepacket spread out temporally and spatially [17,20]. Note that, A(r,ω), normalized here is actually closely related to the amplitude spectrum of $V_{0}(\omega )$ and will be taken into account in Section 3.2. Applying inverse Fourier transform (IFT) to Equation (4), the sensor signal $v_{0}(t)$ of nonlinear-dispersion can be calculated in the time domain.

### 2.2. Linear-Dispersion and Non-Dispersion Effects

The narrowband excitation signal of Lamb waves is normally given as an amplitude modulated harmonic [28]

(5a) $$v_{a}(t)=m(t)e^{i\omega_{c}t}$$

(5b) $$V_{a}(\omega )=M(\omega -\omega_{c})$$

where $v_{a}(t)$ is the excitation signal in the time domain. m(t) is the amplitude modulation function specifying the envelope of $v_{a}(t)$, $M(\omega )=\int m(t)e^{-i\omega t}dt$ is the Fourier transform (FT) result of m(t) and the carrier frequency $\omega_{c}$ corresponds to the central angular frequency of $v_{a}(t)$.

To further deduce the time-domain sensor signal $v_{lin}(t)$ of linear-dispersion from Equation (4), the corresponding wavenumber can be defined by linearizing $K_{0}(\omega )$ as its first-order Taylor series expansion around $\omega_{c}$ [28]

(6) $$\begin{array}{cc} K_{lin}(\omega ) & =k_{0}+k_{1}(\omega -\omega_{c})\\ & =k_{1}\omega +(k_{0}-k_{1}\omega_{c}) \end{array}$$

where $K_{lin}(\omega )$ is the linearly-dispersive wavenumber relation of $v_{lin}(t)$, $k_{0}=\omega_{c}/c_{p}(\omega_{c})$, $k_{1}=dK_{0}(\omega )/d\omega {|}_{\omega =\omega_{c}}=1/c_{g}(\omega_{c})$ and

(7) $$c_{p\_lin}(\omega )=c_{g}(\omega_{c})/\left\{1+\omega_{c}/\omega \left[c_{g}(\omega_{c})/c_{p}(\omega_{c})-1\right]\right\},\;c_{g\_lin}(\omega )=c_{g}(\omega_{c})$$

where $c_{p\_lin}(\omega )$ and $c_{g\_lin}(\omega )$ are the phase and group velocities under linear-dispersion, respectively. Supposing $k_{0}\neq k_{1}\omega_{c}$, i.e., $\omega_{c}/c_{p}(\omega_{c})\neq \omega_{c}/c_{g}(\omega_{c})$ and $c_{p}(\omega_{c})\neq c_{g}(\omega_{c})$ without losing generality, it can be seen from Equation (7) that $c_{p\_lin}(\omega )$ varies with ω while $c_{g\_lin}(\omega )$ is a constant $c_{g}(\omega_{c})$.

Inserting Equations (5a), (5b) and (6) into Equation (4) and applying IFT, $v_{lin}(t)$ is obtained with shifting property of FT as

(8) $$\begin{array}{cc} v_{lin}(t) & =\frac{1}{2\pi }\int M(\omega -\omega_{c})e^{-i\left[k_{0}+k_{1}(\omega -\omega_{c})\right]r+i\omega t}d\omega \\ & =\frac{e^{i\omega_{c}t-ik_{0}r}}{2\pi }\int M(\omega )e^{-i\omega k_{1}r+i\omega t}d\omega \\ & =m(t-k_{1}r)e^{i\omega_{c}t-ik_{0}r}\\ & =v_{a}(t-k_{1}r)e^{ir(k_{1}\omega_{c}-k_{0})} \end{array}$$

In Equation (8), $v_{lin}(t)$ is simply a $v_{a}(t)$ delayed by its travelling time $r/c_{g}(\omega_{c})$ with an extra factor $e^{ir(k_{1}\omega_{c}-k_{0})}$. Since $k_{0}\neq k_{1}\omega_{c}$, $e^{ir(k_{1}\omega_{c}-k_{0})}\neq 1$. The initial phase of carrier frequency $\omega_{c}$ of $v_{lin}(t)$ is uniformly shifted by $r(k_{1}\omega_{c}-k_{0})=\omega_{c}\left[r/c_{g}(\omega_{c})-r/c_{p}(\omega_{c})\right]$ without any signal envelope deformation. The main dispersion problem of broadening and disturbing the travelling wavepacket in the original nonlinearly-dispersive signal $v_{0}(t)$ will no longer occur in $v_{lin}(t)$ under linear-dispersion.

As a special case, when $k_{0}=k_{1}\omega_{c}$ in Equation (6), the non-dispersive relation $K_{non}(\omega )$ is satisfied, i.e.,

(9) $$K_{non}(\omega )=k_{1}\omega$$

Both phase and group velocities of non-dispersion are a constant $c_{g}(\omega_{c})$. From Equation (8), the non-dispersive time-domain sensor signal $v_{non}(t)=v_{a}(t-k_{1}r)$, indicating that $v_{non}(t)$ is only a time-delayed version of $v_{a}(t)$ and free of any dispersion effect.

Noted that, $K_{non}(\omega )$ and $K_{lin}(\omega )$ are practically the results of linearizing $K_{0}(\omega )$ at the original point and at the point of $K_{0}(\omega_{c})$, respectively. With a non-zero offset $k_{0}-k_{1}\omega_{c}$ as compared to $K_{non}(\omega )$, $K_{lin}(\omega )$, though a straight line about ω, does not possess a strictly-defined linear relation with respect to ω. This is why $K_{lin}(\omega )$ is referred in this study as a specific dispersive wavenumber relation, i.e., the linearly-dispersive wavenumber relation, under which the dispersion effect of changing the initial phase of carrier frequency $\omega_{c}$ of $v_{lin}(t)$ can be generally disregarded.

### 2.3. Numerical Simulation

**Figure 1:**
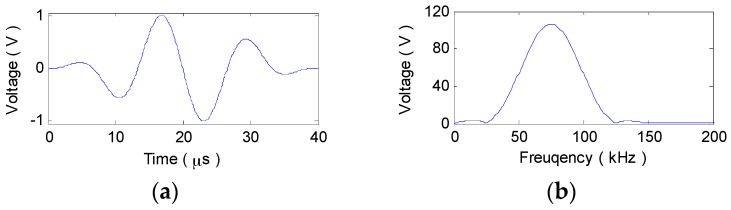
Excitation signal (*f_c_* = 75 kHz). (**a**) Waveform; (**b**) Spectrum.

To validate the above analysis, a numerical simulation on A_0_ or S_0_ mode signals traveling for 30 cm in a 2024 aluminum plate with the thickness of 2 mm are conducted. Using the plate material parameters in Table 1, the original nonlinear-dispersion wavenumber relations $K_{0}(\omega )$ of the two fundamental modes are theoretically derived from the Rayleigh-Lamb dispersion equation [23,28,30]. With Equations (6) and (9), the linearized wavenumber relations $K_{lin}(\omega )$ and $K_{non}(\omega )$ can be calculated. As shown in Figure 1, the narrowband excitation signal is a modulated 3-cycle sine burst, the central frequency $f_{c}$ of which is selected as 75 kHz and 900 kHz to maximize the dispersion effect on A_0_ and S_0_ mode signals, respectively. Taking $K_{0}(\omega )$ in Equation (4) as the original nonlinear wavenumber relation or the two kinds of linearized ones and applying IFT, the sensor signal $v_{0}(t)$, $v_{lin}(t)$ or $v_{non}(t)$ can be synthesized.

**Figure 2:**
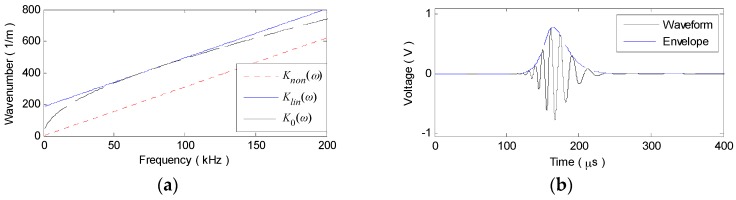

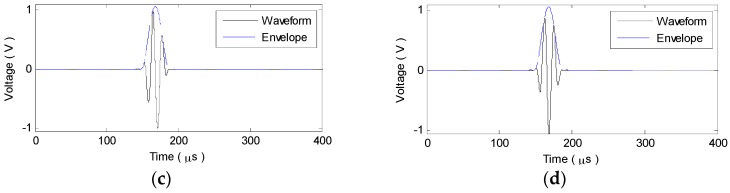
Simulation results of the A_0_ mode signal. (**a**) $K_{0}(\omega )$, $K_{lin}(\omega )$ and $K_{non}(\omega )$; (**b**) $v_{0}(t)$; (**c**) $v_{non}(t)$; (**d**) $v_{lin}(t)$.

The simulation results of the A_0_ mode signal are shown in Figure 2. As Figure 2a illustrates, the theoretical wavenumber relations $K_{0}(\omega )$ is a curve, showing the nonlinear-dispersion property. While the linearized ones $K_{lin}(\omega )$ and $K_{non}(\omega )$ are the two parallel straight lines and a constant offset exists between them. In contrast to $K_{lin}(\omega )$, $K_{non}(\omega )$ passing through the original point exhibits the more ideal linear relation with ω. Due to the nonlinear $K_{0}(\omega )$, the wavepacket in $v_{0}(t)$ is no longer a 3-cycle windowed toneburst and the time duration increases from 40 μs (seen in Figure 1a) to about 150 μs with its waveform disturbed and its amplitude decreased, as shown in Figure 2b. With linearized $K_{lin}(\omega )$ and $K_{non}(\omega )$, both the wavepackets in $v_{lin}(t)$ and $v_{non}(t)$ are exempt from expansion and distortion, as Figure 2c,d illustrate. Note that, the wavepacket in $v_{lin}(t)$ remains a 3-cycle toneburst, but undergoes some waveform alteration in changing the relative sites and amplitudes of the three crests and troughs, as compared with that of $v_{non}(t)$ or the excitation signal. This is caused by the initial-phase shift to the carrier wave of the wavepacket. Clearly, it is the only effect that the linear-dispersion brings about and can be generally ignored.

**Figure 3:**
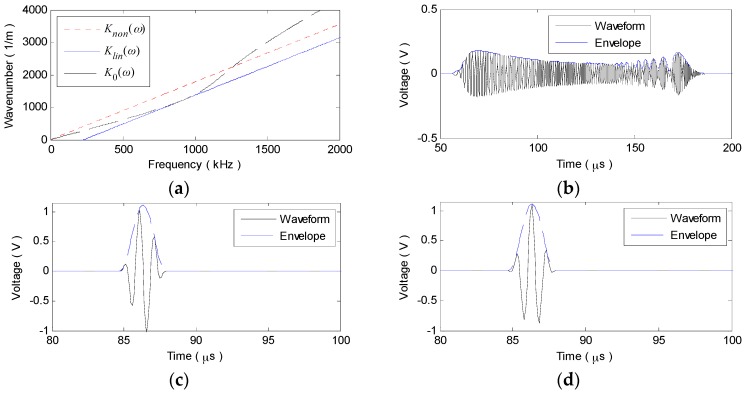
Simulation results of the S_0_ mode signal. (**a**) $K_{0}(\omega )$, $K_{lin}(\omega )$ and $K_{non}(\omega )$; (**b**) $v_{0}(t)$; (**c**) $v_{non}(t)$; (**d**) $v_{lin}(t)$.

The similar simulation results of the S_0_ mode signal can be obtained, as shown in Figure 3. Under a much more nonlinear $K_{0}(\omega )$ (seen in Figure 3a), the wavepacket in $v_{0}(t)$ is largely extended to the tanglesome waveform with its amplitude severely decreased, as shown in Figure 3b. Whereas, the wavapacket shapes remain unchanged in both $v_{lin}(t)$ and $v_{non}(t)$, as illustrated in Figure 3c,d.

## 3. Linearly-Dispersive or Non-Dispersive Signal Construction

As discussed above, it is the nonlinear wavenumber $K_{0}(\omega )$ that causes the wavepacket elongation and distortion in $v_{0}(t)$. The nonlinear-dispersion phenomenon can be well avoided if $K_{0}(\omega )$ is linearized to $K_{lin}(\omega )$ or $K_{non}(\omega )$. This could exactly lead to the purpose of linearly-dispersive signal construction (LDSC) and non-dispersive signal construction (NDSC). In signal construction, the signal $v_{lin}(t)$ or $v_{non}(t)$ is respectively constructed from the original nonlinearly-dispersed one $v_{0}(t)$ using $K_{0}(\omega )$. By doing this, the nonlinear wavenumber is practically linearized for dispersion compensation. Compared with the traditional signal domain transform methods [23,30], LDSC or NDSC is only performed in the time-frequency domain and a transfer to the distance-wavenumber domain is not required. In the section, after the basic signal construction principle is investigated, the numerical realization of LDSC and NDSC is discussed, in which both the signal waveform maintenance and the amplitude spectrum preservation are particularly concerned during the signal construction process. A comparison is also performed with the typical signal domain transform methods of TDDM and TDDT.

### 3.1. Basic Principle of Signal Construction

In Lamb wave detection, the traveling distance r is probably unknown especially for a damage scattered signal. $v_{lin}(t)$ or $v_{non}(t)$ can not be directly synthesized using Equation (4), as mentioned in Section 2.3. The basic signal construction principle is thus fruitfully explored here to calculate $v_{lin}(t)$ or $v_{non}(t)$ based on the sensing model without requiring r. Since the excitation signal $V_{a}(\omega )$ is known in a priori, from Equation (4), the crucial problem in signal construction is how to pursue the corresponding phase-delay factor. Its general expression can be rewritten in a composite function [28,30]

(10) $$E(r,\omega )=E\left[r,K(\omega )\right]=e^{-ikr}{|}_{k=K(\omega )}$$

where the subfunction is the dispersion relation K(ω) and the generating function $E(r,k)=e^{-ikr}$. For a given r, E(r,k) is only a simple exponential function and irrespective of the exact variation relation of its independent variable k, i.e., K(ω), which implies that E(r,ω) under various dispersion relations is subject to an identical E(r,k).

From Equation (10), E(r,k) and E(r,ω) are the two distinct functions defined in the wavenumber or frequency domain, but share the same functional values. Thus, mathematically, performing the wavenumber-domain interpolation to E(r,k) at the equally-spaced frequency points k=K(ω) will result in E(r,ω), the procedure of which can be mentioned as the construction of E(r,ω). Vice versa, during the so-called deconstruction of E(r,ω), E(r,k) can be obtained by interpolating E(r,ω) with even wavenumber intervals in terms of $\omega =K^{-1}(\omega )$, where $K^{-1}(\omega )$ is the inverse function of K(ω).

**Figure 4:**
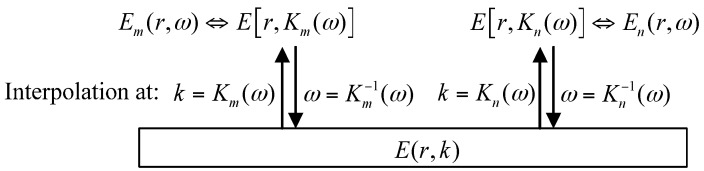
Correlations between the phase-delay factors $E_{m}(r,\omega )$ and $E_{n}(r,\omega )$.

Consider two arbitrary time-domain signals, $v_{n}(t)$ and $v_{m}(t)$ of the same propagation paths but different dispersion relations $K_{m}(\omega )$ and $K_{n}(\omega )$. Their phase-delay factors ($E_{m}(r,\omega )=E\left[r,K_{m}(\omega )\right]$ and $E_{n}(r,\omega )=E\left[r,K_{n}(\omega )\right]$) are correlated to each other on the basis of the identical E(r,k) *via* the above construction and deconstruction processes, as shown in Figure 4, where $K_{m}^{-1}(\omega )$ and $K_{n}^{-1}(\omega )$ are the inverse functions of $K_{m}(\omega )$ and $K_{n}(\omega )$, respectively. This can provide an approach to calculate one of the two factors, $E_{n}(r,\omega )$ for example, when the other variables $E_{m}(r,\omega )$, $K_{m}(\omega )$ and $K_{n}(\omega )$ are given. That is:

(a) After the inverse function $K_{m}^{-1}(\omega )$ is established from $K_{m}(\omega )$, the frequency-domain interpolation is carried out to $E_{m}(r,\omega )$ at $\omega =K_{m}^{-1}(\omega )$ and $E_{m}(r,\omega )$ is deconstructed to E(r,k);

(b) By implementing interpolation to E(r,k) at $k=K_{n}(\omega )$ in the wavenumber domain, the phase-delay factor $E\left[r,K_{n}(\omega )\right]$ under the dispersion relation $K_{n}(\omega )$, i.e., $E_{n}(r,\omega )$ is successfully constructed.

According to the calculating procedure, $E_{n}(r,\omega )$ can be mapped to $E_{m}(r,\omega )$ straightforwardly as

(11) $$E_{n}(r,\omega )=E_{m}\left[r,\Omega_{n}(\omega )\right],\;\Omega_{n}(\omega )=K_{m}^{-1}\left[K_{n}(\omega )\right]$$

where $\Omega_{n}(\omega )$ is the interpolation mapping sequence from $E_{m}(r,\omega )$ to $E_{n}(r,\omega )$. Equation (11) suggests that, $E_{n}(r,\omega )$ can be directly attained from $E_{m}(r,\omega )$ through only one time frequency-domain interpolation with $\Omega_{n}(\omega )$, so that E(r,k) and the wavenumber-domain interpolation for it is not needed. Both the interpolation error and computation cost can be thus decreased. Furthermore, the transform for the phase-delay factor between the frequency and wavenumber domains, implemented in TDDM or TDDT, is not required.

Inserting Equation (11) into Equation (4) and applying IFT, the basic formula of signal construction is established as

(12) $$v_{n}(t)=\frac{1}{2\pi }\int V_{a}(\omega )E_{m}\left[r,\Omega_{n}(\omega )\right]e^{i\omega t}d\omega$$

Note that, the travelling distance r, already presented in the phase-delay factor $E_{m}(r,\omega )$, is not expressly needed during the construction of $v_{n}(t)$.

### 3.2. Numerical Realization of LDSC and NDSC

With $K_{m}(\omega )$ corresponding to $K_{0}(\omega )$, and $K_{n}(\omega )$ to $K_{lin}(\omega )$ or $K_{non}(\omega )$, the numerical realization of LDSC or NDSC can be expected based on Equation (12), respectively. Note that, since the aforementioned signals in different domains are stored as discrete ones in reality, FT and IFT are respectively replaced by Fast Fourier transform (FFT) and Inverse Fast Fourier transform (IFFT) in the following discussion.

Taking LDSC for instance, from Equation (12), $K_{0}(\omega )$, $K_{lin}(\omega )$ and the phase-delay factor $E_{0}(r,\omega )=e^{-iK_{0}(\omega )r}$ should be determined to calculate $v_{lin}(t)$. $K_{0}(\omega )$ can be theoretically derived from the Rayleigh-Lamb dispersion equation using the structure material parameters. Then, $K_{lin}(\omega )$ is decided by linearizing $K_{0}(\omega )$ with Equation (6). As for the phase-delay factor $E_{0}(r,\omega )$, Equation (2) indicates that, $E_{0}(r,\omega )$ is equivalent to the transfer function H(ω) with the amplitude A(r,ω) normalized. H(ω) is the frequency spectrum of the temporal impulse response h(t). Hence, H(ω) as well as $E_{0}(r,\omega )$ can be obtained by applying FFT to h(t). h(t) can be obtained under impulse or step pulse excitation [20].

With Equations (4) and (12) and using the convolution property of FT, $v_{lin}(t)$ can be finally calculated as

(13) $$\begin{array}{cc} v_{lin}(t) & =\mathrm{IFFT}\left\{\left[V_{a}(\omega )\right]\cdot H\left[\Omega_{lin}(\omega )\right]\right\}\\ & =v_{a}(t)*\mathrm{IFFT}\left\{H\left[\Omega_{lin}(\omega )\right]\right\} \end{array}$$

where IFFT[ ] and ∗ denote IFFT and convolution operations, respectively. $\Omega_{lin}(\omega )$ is the interpolation mapping sequence in LDSC,

(14) $$\Omega_{lin}(\omega )=K_{0}^{-1}\left[K_{lin}(\omega )\right]$$

Analogously, for the numerical realization of NDSC, it can be derived from Equations (13) and (14) that

(15) $$v_{non}(t)=v_{a}(t)*\mathrm{IFFT}\left\{H\left[\Omega_{non}(\omega )\right]\right\}$$

(16) $$\Omega_{non}(\omega )=K_{0}^{-1}\left[K_{non}(\omega )\right]$$

where $\Omega_{non}(\omega )$ is the interpolation mapping sequence in NDSC. Note that, $K_{0}(\omega )$ is assumed to be a monotonic function to guarantee the existence of $\Omega_{lin}(\omega )$ or $\Omega_{non}(\omega )$.

Equations (13) and (15) are the calculation formulas of LDCS and NDSC, respectively. Due to the frequency-domain interpolation in signal construction, besides the dispersion relation, the other signal properties, especially the signal waveform and amplitude spectrum could be undesirably changed at the same time. Therefore, two important things should be considered:

(a) Signal waveform maintenance. Apparently, the nonlinear-dispersion relation $K_{0}(\omega )$ is just decided by $E_{0}(r,\omega )$ and irrespective of the narrowband excitation signal $V_{a}(\omega )$. In LDSC or NDSC, the broadband excitation of impulse or step pulse, rather than the commonly-used narrowband excitation, is adopted to acquire h(t), so that $E_{0}(r,\omega )$ is individually obtained as H(ω) and the frequency-domain interpolation for it can be expediently executed for dispersion removal without any influence on $V_{a}(\omega )$, as expressed by Equation (13) or (15). As a result, the waveform maintenance for $v_{lin}(t)$ or $v_{non}(t)$ can be achieved.

Conversely, when the narrowband excitation is used to acquire the single mode signal $v_{0}(t)$ as usual, h(t) and therefore, H(ω) cannot be pursued straightforwardly, but implicitly given together with $V_{0}(\omega )$. The frequency-domain interpolation in Equation (13) or (15) has to be implemented on $V_{0}(\omega )$, instead of H(ω). That is

(17) $$\begin{array}{cc} v_{lin}^{\prime }(t) & =\mathrm{IFFT}\left\{V_{0}\left[\Omega_{lin}(\omega )\right]\right\}\\ & =\mathrm{IFFT}\left\{V_{a}\left[\Omega_{lin}(\omega )\right]\cdot H\left[\Omega_{lin}(\omega )\right]\right\} \end{array}$$

(18) $$\begin{array}{cc} v_{non}^{\prime }(t) & =\mathrm{IFFT}\left\{V_{0}\left[\Omega_{non}(\omega )\right]\right\}\\ & =\mathrm{IFFT}\left\{V_{a}\left[\Omega_{non}(\omega )\right]\cdot H\left[\Omega_{non}(\omega )\right]\right\} \end{array}$$

In Equation (17) or (18), because of the entire interpolation on $V_{0}(\omega )$, the frequency content of $V_{a}(\omega )$ is disarranged as $V_{a}\left[\Omega_{lin}(\omega )\right]$ or $V_{a}\left[\Omega_{non}(\omega )\right]$, making $v_{a}(t)$ dominated by other frequency components. The waveform deformation, similar to the frequency-shifting phenomenon is then produced to $v_{a}(t)$ and the final constructed signal [30]. Additionally, the more $\Omega_{lin}(\omega )$ or $\Omega_{non}(\omega )$ differs from ω, the more serious deformation will occur to $v_{lin}^{\prime }(t)$ or $v_{non}^{\prime }(t)$. Note that, Equation (17) is actually the calculation formula of LM [28]. Since $K_{lin}(\omega )$ is the local linear approximation of the original dispersive one K(ω) with respect to the central frequency $\omega_{c}$, the mapped frequency point $\Omega_{lin}(\omega )$ deviates much less from the former one ω, as compared with $\Omega_{non}(\omega )$. The waveform deformation in LM is very slight and can be disregarded [28,29].

(b) Amplitude spectrum preservation. With dispersion property only involved in the phase-delay factor $E_{0}(r,\omega )$, the other crucial signal characteristics, including the signal amplitude and physical damage sensitivity are mainly dependent on the amplitude spectrum A(r,ω). Hence, A(r,ω) normalized and neglected in the above discussion should be treated carefully during LDSC or NDSC. Taking A(r,ω) into account, from Equations (1), (2) and (13), $v_{lin}(t)$, for example is actually constructed as

(19) $$v_{lin}(t)=\mathrm{IFFT}\left\{V_{a}(\omega )\cdot A\left[r,\Omega_{lin}(\omega )\right]\cdot E_{0}\left[r,\Omega_{lin}(\omega )\right]\right\}$$

Equation (19) indicates that, A(r,ω) is diverged to $A\left[r,\Omega_{lin}(\omega )\right]$ and should be restored. However, unfortunately, A(r,ω) is a very complicated function of r and ω. Moreover, $v_{0}(t)$ normally consists of multiple wavepackets with different unknown r. It is extremely hard to exactly calculate and correct A(r,ω).

Unlike $K_{non}(\omega )$, $K_{lin}(\omega )$ is established by linearizing $K_{0}(\omega )$ at $K_{0}(\omega_{c})$ and is actually the local linear approximation of $K_{0}(\omega )$ around the central frequency $\omega_{c}$. Even $K_{lin}(\omega_{c})=K_{0}(\omega_{c})$ at $\omega_{c}$. From Equation (14), the mapped frequency point $\Omega_{lin}(\omega )$ during the interpolation will deviate much less from the former one ω, especially within the frequency range of $v_{0}(t)$. Because of this, the influence of LDSC on A(r,ω) is so slight that can be nearly neglected. While in NDSC, with the non-zero offset $k_{0}-k_{1}\omega_{c}$ between $K_{non}(\omega )$ and $K_{lin}(\omega )$, $\Omega_{non}(\omega )$ differs much more from ω and A(r,ω) is greatly varied.

This makes LDSC more preferable in some adverse situations, such as the serious energy loss during Lamb wave travelling in complex structures or the weak flaws in tested structures. In such situations, to ensure sufficient SNR and the damage sensitivity of Lamb wave signals, the highest possible A(r,ω) within the signal bandwidth is usually selected by adjusting $\omega_{c}$ of $V_{0}(\omega )$ and needs to be mostly unchangeable. It is clear that, with the signal waveform retained in the two signal construction approaches, the amplitude spectrum can be only preserved in LDSC.

### 3.3. Comparison with TDDM and TDDT

LDSC and NDSC are largely analogous to the signal domain transform methods. It is necessary to conduct a comparison with the typical signal domain transform methods, i.e., TDDM and TDDT, the calculation formulas of which are respectively given as [23,30]

(20) $$\begin{array}{cc} \tilde{v}(r) & =\mathrm{IFFT}\left\{V_{0}\left[\Omega (k)\right]\right\}\\ & =\mathrm{IFFT}\left\{V_{a}\left[\Omega (k)\right]\right\}*\mathrm{IFFT}\left\{H\left[\Omega (k)\right]\right\} \end{array}$$

(21) $$\begin{array}{cc} v(r) & =\mathrm{IFFT}\left\{V_{a}\left[\Omega_{non}(k)\right]\right\}*\mathrm{IFFT}\left\{H\left[\Omega (k)\right]\right\}\\ & =v_{a}(r)*\mathrm{IFFT}\left\{H\left[\Omega (k)\right]\right\} \end{array}$$

where $\Omega (k)=K_{0}^{-1}(\omega )$, $\Omega_{non}(k)=K_{non}^{-1}(\omega )$, the distance-domain excitation signal $v_{a}(r)=\mathrm{IFFT}\left\{V_{a}\left[\Omega_{non}(k)\right]\right\}$ and $v_{a}(r)=v_{a}\left[c_{g}(\omega_{c})t\right]$ [30], $\tilde{v}(r)$ and v(r) are the results of TDDM and TDDT, respectively.

To facilitate the comparison, the Formulas (13), (15), (20) and (21) are rewritten as

(22) $$\begin{array}{cc} v_{lin}(t) & =v_{a}(t)*\mathrm{IFFT}\left\{A\left[r,\Omega_{lin}(\omega )\right]\cdot E_{0}\left[r,\Omega_{lin}(\omega )\right]\right\}\\ & =v_{a}(t)*\mathrm{IFFT}\left\{A\left[r,\Omega_{lin}(\omega )\right]\cdot E_{lin}\left(r,\omega \right)\right\} \end{array}$$

(23) $$\begin{array}{cc} v_{non}(t) & =v_{a}(t)*\mathrm{IFFT}\left\{A\left[r,\Omega_{non}(\omega )\right]\cdot E_{0}\left[r,\Omega_{non}(\omega )\right]\right\}\\ & =v_{a}(t)*\mathrm{IFFT}\left\{A\left[r,\Omega_{non}(\omega )\right]\cdot E_{non}\left(r,\omega \right)\right\} \end{array}$$

(24) $$\begin{array}{cc} \tilde{v}(r) & =\mathrm{IFFT}\left\{V_{a}\left[\Omega (k)\right]\right\}*\mathrm{IFFT}\left\{A\left[r,\Omega (k)\right]\cdot E_{0}\left[r,\Omega (k)\right]\right\}\\ & =\mathrm{IFFT}\left\{V_{a}\left[\Omega (k)\right]\right\}*\mathrm{IFFT}\left\{A\left[r,\Omega (k)\right]\cdot E_{0}(r,k)\right\} \end{array}$$

(25) $$\begin{array}{cc} v(r) & =v_{a}(r)*\mathrm{IFFT}\left\{A\left[r,\Omega (k)\right]\cdot E_{0}\left[r,\Omega (k)\right]\right\}\\ & =v_{a}(r)*\mathrm{IFFT}\left\{A\left[r,\Omega (k)\right]\cdot E_{0}(r,k)\right\} \end{array}$$

where $E_{lin}\left(r,\omega \right)=E_{0}\left[r,\Omega_{lin}(\omega )\right]$, $E_{non}\left(r,\omega \right)=E_{0}\left[r,\Omega_{non}(\omega )\right]$ and $E_{0}\left(r,k\right)=E_{0}\left[r,\Omega (k)\right]$.

It can be found that:

(a) Both the signal construction methods and the signal domain transform ones are essentially a kind of spectral warping process [32]. In the process, as expressed by the similar Equations (22)–(25), each frequency point ω is separately interpolated or mapped as another one, i.e., $\Omega_{lin}(\omega )$, $\Omega_{non}(\omega )$ or Ω(k), to fulfill nonlinear frequency rescaling on $E_{0}(r,\omega )$. As a result, the spectral chaos in $E_{0}(r,\omega )$ produced by the nonlinear $K_{0}(\omega )$ can be restored for dispersion elimination.

(b) The basic dispersion compensation idea of the two kinds of methods is different. Equations (22)–(25) imply that the critical point for dispersion compensation is how to remove the effect of nonlinear wavenumber $K_{0}(\omega )$ on the phase-delay factor $E_{0}(r,\omega )$. In LDSC or NDSC, as expressed by Equations (22) and (23), the effect is eliminated by replacing $E_{0}(r,\omega )$ with the newly-calculated $E_{lin}\left(r,\omega \right)$ or $E_{non}\left(r,\omega \right)$. This is equivalent to linearizing the nonlinear $K_{0}(\omega )$ in $E_{0}(r,\omega )$ as $K_{lin}(\omega )$ or $K_{non}(\omega )$, respectively. Thus, the nonlinear wavenumber linearization is the basis of dispersion compensation in LDSC and NDSC. While in TDDM or TDDT, since the generating function $E_{0}(r,k)$ is irrelevant to $K_{0}(\omega )$ and free of nonlinear $K_{0}(\omega )$-induced dispersion effect, $E_{0}(r,\omega )$ is deconstructed to $E_{0}(r,k)$ for dispersion removal in Equations (24) and (25). Since $E_{0}(r,\omega )$ and $E_{0}(r,k)$ correspond to the distinct domains of time-frequency and distance-wavenumber, the conception of signal domain transferring is introduced in TDDM and TDDT.

(c) Different from TDDM expressed by Equation (20), H(ω), rather than $V_{0}(\omega )$ is acquired in Equation (13), (15) or (21) to avoid the disarrangement of excitation signal spectra. Therefore, signal waveform maintenance is not regarded in TDDM, but in LDSC, NDSC and TDDT.

(d) Comparing Equations (15) and (21), $v_{non}(t)$ and v(r) can be transferred to each other with the simple variable scaling relation $r=c_{g}(\omega_{c})t$. Thus, TDDT and NDSC are actually equivalent. The same problem of significantly changing the amplitude spectrum would occur in TDDT. Generally, among the four dispersion compensation approaches, only in LDSC can preservation of both signal waveform and amplitude spectrum be accomplished.

## 4. High Spatial Resolution Imaging Based on LDSC or NDSC

**Figure 5:**
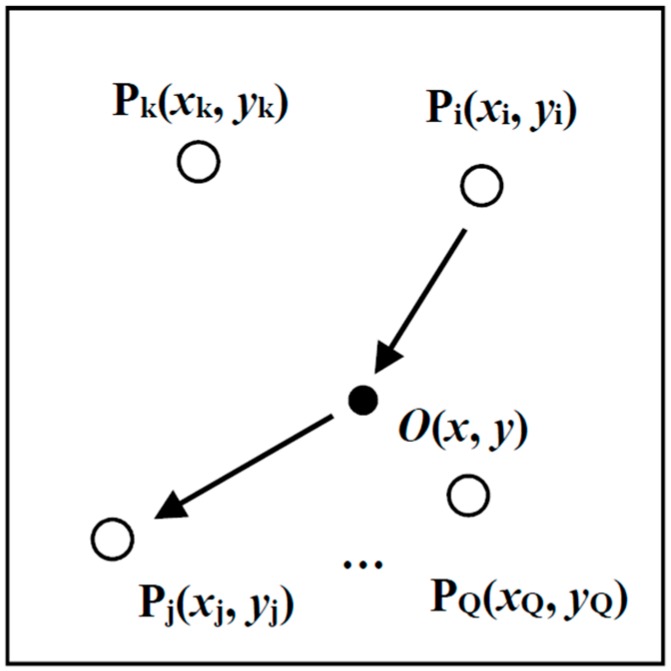
Illustration of the delay-and-sum imaging.

LDSC or NDSC can be performed as a general signal processing approach for Lamb wave detection. In this section, the two signal construction methods are applied associated with the delay-and-sum algorithm for high spatial resolution damage imaging. Delay-and-sum is a commonly-employed Lamb wave imaging algorithm of sparse PZT arrays [13,14]. The imaging algorithm can be illustrated by Figure 5, where the monitored structure is integrated with a sparse transducer array of Q(Q≥3) PZT wafers. For a PZT pair $P_{i-j}(i\neq j;i=1,2,\cdot \cdot \cdot ,Q;j=1,2,\cdot \cdot \cdot ,Q)$ composed of $P_{i}$ at $\left(x_{i},y_{i}\right)$ and $P_{j}$ at $\left(x_{j},y_{j}\right)$, the relevant propagation time with respect to an arbitrary point O at (x,y) can be geometrically decided assuming that only one Lamb wave mode exists with a constant group velocity $c_{g}(\omega_{c})$ [13]

(26) $$t_{ij}(x,y)=\left[\sqrt{{\left(x_{i}-x\right)}^{2}+{\left(y_{i}-y\right)}^{2}}+\sqrt{{\left(x_{j}-x\right)}^{2}+{\left(y_{j}-y\right)}^{2}}\right]/c_{g}(\omega_{c})$$

If $s_{ij}(t)$ is the scattered signal measured by $P_{i-j}$, then $s_{ij}(t_{ij}(x,y))$ is related to the amplitude of the signal scattered from the point O. All the scattered signals measured by PZT pairs $P_{i-j}(i\neq j;1\leq i,j\leq Q)$ are time-delayed and summarized to get an averaged energy of point *O*. That is

(27) $$E(x,y)={\left[\frac{2}{Q(Q-1)}\sum_{i=1}^{Q}\sum_{j=i+1}^{Q}s_{ij}\left(t_{ij}\left(x,y\right)\right)\right]}^{2}$$

With the energy E(x,y) of each point normalized and grey-scaled, a damage image over the whole structure can be generated. The local spots in the image with strong intensities will probably correspond to the actual defects.

The delay-and-sum imaging method is a simple but effective method that can automatically focus the damage scattered signal measure by every PZT pair to any real flaw points. However, the nonlinear-dispersion could affect the scattered signal to produce an inferior resolution imaging result, from which neither a single flaw nor multiple ones can be easily identified. To remove the dispersion influence, LDSC or NDSC is firstly introduced for $s_{ij}(t)$ during damage imaging. The pixel value of point O is then calculated as

(28) $$E_{lin}(x,y)={\left[\frac{2}{Q(Q-1)}\sum_{i=1}^{Q}\sum_{j=i+1}^{Q}s_{ij\_lin}\left(t_{ij}\left(x,y\right)\right)\right]}^{2}$$

(29) $$E_{non}(x,y)={\left[\frac{2}{Q(Q-1)}\sum_{i=1}^{Q}\sum_{j=i+1}^{Q}s_{ij\_non}\left(t_{ij}\left(x,y\right)\right)\right]}^{2}$$

where $s_{ij\_lin}(t)$ and $s_{ij\_non}(t)$ are the LDSC-processed and NDSC-processed $s_{ij}(t)$, respectively.

In Equations (28) and (29), since each dispersed wavepacket in $s_{ij}(t)$ is recovered *via* LDSC or NDSC in $s_{ij\_lin}(t)$ or $s_{ij\_non}(t)$, a great improvement in the spatial resolution of damage imaging can be made. The capacity of Lamb wave adjacent multi-damage or quantitative imaging is then significantly enhanced.

## 5. Experimental and Numerical Validations

### 5.1. Imaging Experiment of Adjacent Dual Damages

#### 5.1.1. Experimental Setup

**Figure 6:**
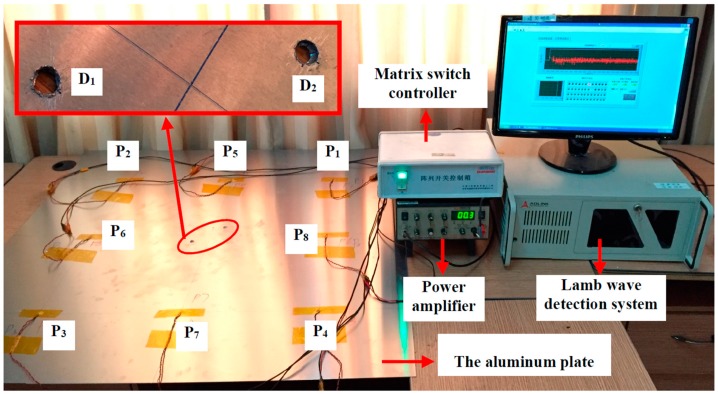
Experimental setup.

To verify the proposed signal construction and high spatial resolution imaging methods, an experiment is arranged on a 1000 mm × 1000 mm × 1.5 mm 2024 aluminum plate, which is largely used in aerospace structures. The material properties of the plate are given in Table 1. In the experimental validation, the adjacent dual damage imaging is carried out. As illustrated in Figure 6, the overall experiment setup is composed of Lamb wave detection system, matrix switch controller, power amplifier and the aluminum plate. Lamb wave detection system can generate Lamb wave signals, amplify and collect sensor signals. The matrix switch controller controls the working sequence of all PZT pairs and the power amplifier is applied to amplify the excitation signal to enlarge the monitoring area of the plate.

**Figure 7:**
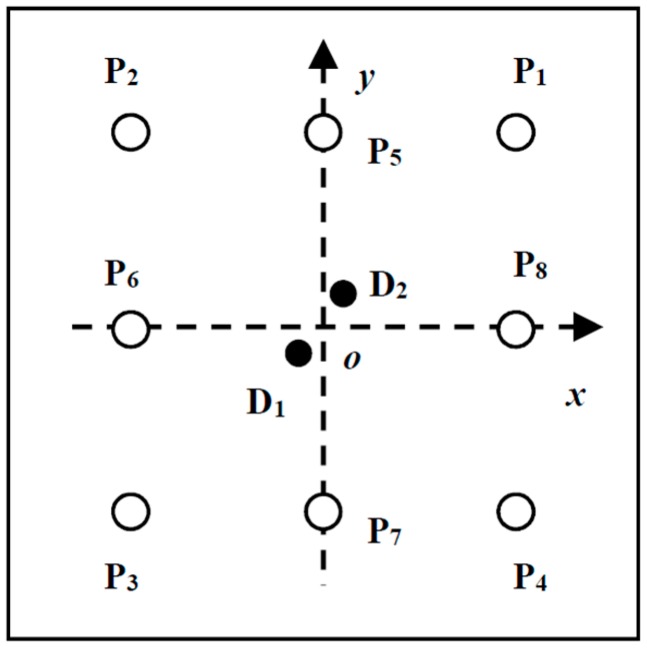
Distribution of the PZT array and damages in the monitored region.

To monitor the entire plate, eight PZT wafers $P_{1}$~$P_{8}$ (PZT-5, 8 mm in diameter and 0.5 mm in thickness) are deployed to form a square transducer array, as shown in Figure 6. The adjacent dual defects D_1_ and D_2_ are produced as the two closely-located through-holes with the diameter of 9 mm by an electrodrill. Figure 7 shows the distribution of the PZT array and dual damages in the monitored region. Their exact positions in the orthogonal coordinate (seen in Figure 7) are listed in Table 2.

#### 5.1.2. Compensation Effect on the Sensor Signals

**Figure 8:**
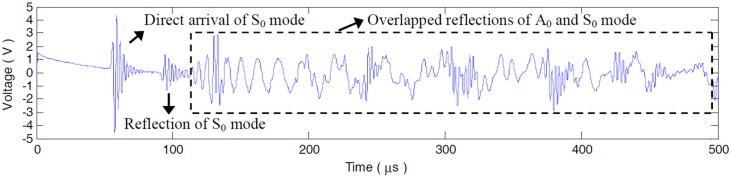
Step pulse response $g_{26}(t)$ of $P_{2-6}$.

**Figure 9:**
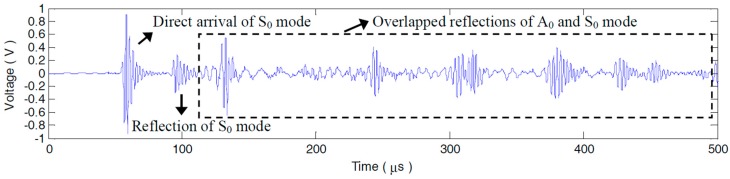
Impulse response $h_{26}(t)$ of $P_{2-6}$.

**Figure 10:**
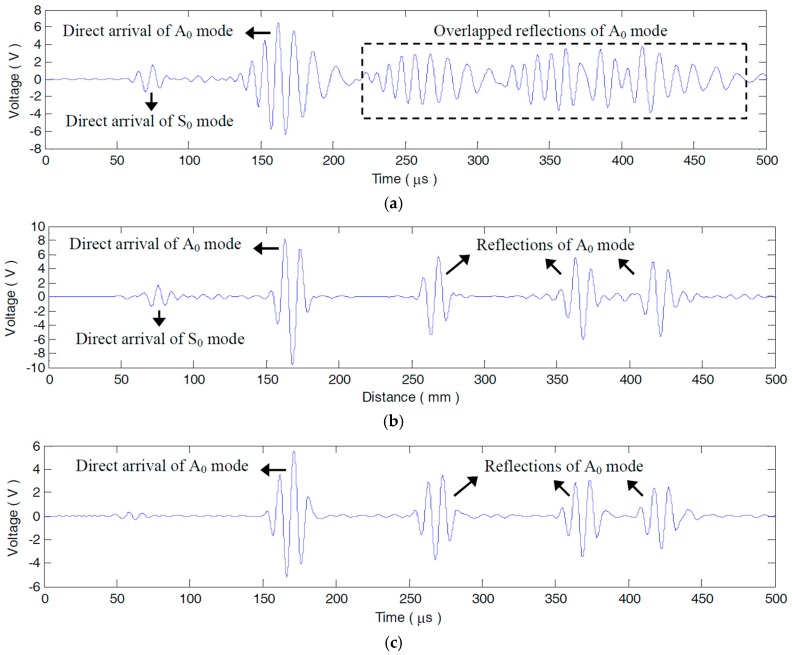

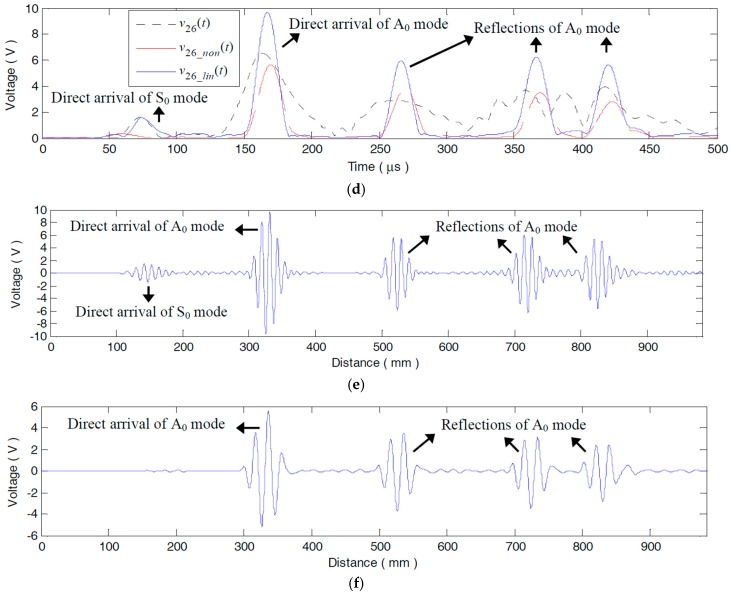
Original extracted and processed A_0_ mode dominated sensor signals measured by $P_{2-6}$. (**a**) Original extracted sensor signal $v_{26}(t)$ from (**b**) LDSC-processed result $v_{26\_lin}(t)$ of $v_{26}(t)$; (**c**) NDSC-processed result $v_{26\_non}(t)$ of $v_{26}(t)$; (**d**) Envelopes of $v_{26}(t)$, $v_{26\_lin}(t)$ and $v_{26\_non}(t)$; (**e**) TDDM-processed result ${\tilde{v}}_{26}(r)$ of $v_{26}(t)$; (**f**) TDDT-processed result $v_{26}(r)$ of $v_{26}(t)$.

A step pulse excitation signal with the raising time of 0.25 μs is produced by the Lamb wave detection system to acquire the impulse response of each PZT pair. By convoluting the impulse response with a 3-cycle sine burst centered at 90 kHz, the desired sensor signals dominated by A_0_ mode can be conveniently extracted. After the derivation calculus of the measured step pulse response $g_{26}(t)$ (seen in Figure 8) measured by $P_{2-6}$ without any defects, the impulse response $h_{26}(t)$ is obtained, as Figure 9 shows. It can be seen that, $g_{26}(t)$ and $h_{26}(t)$ are extremely complicated by multi-mode and dispersion characteristics. What can be recognized from the two broadband responses is merely the S_0_ mode direct arrival and boundary reflection. The following A_0_ or S_0_ mode wavepackets are severely overlapped with each other, as Figure 8 and Figure 9 illustrate. The sensor signal $v_{26}(t)$ extracted from $h_{26}(t)$ is shown in Figure 10a. The A_0_ mode direct arrival in $v_{26}(t)$ distinctly spreads out. Except for the first reflected wavepacket of A_0_ mode near 250 μs in $v_{26}(t)$, the other reflections after 310 μs are badly expanded and superposed. The signal resolution is grievously decreased. Note that, with less dispersion, the direct arrival of S_0_ mode is in a compact wavepacket (seen in the interval 50–100 μs in Figure 10a).

**Figure 11:**
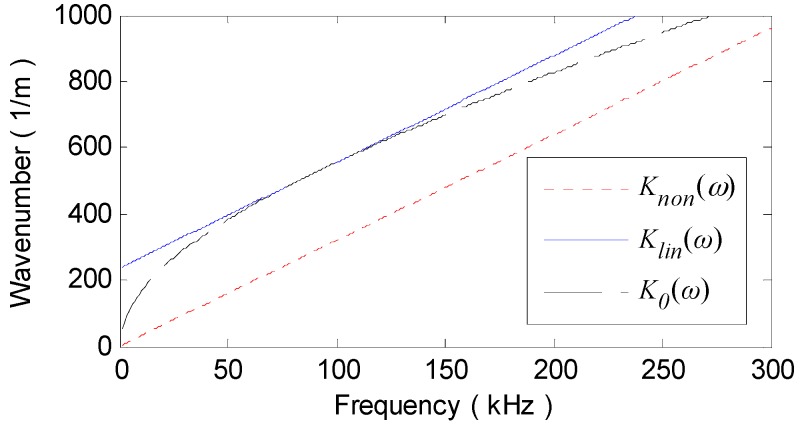
$K_{0}(\omega )$, $K_{lin}(\omega )$ and $K_{non}(\omega )$.

**Figure 12:**
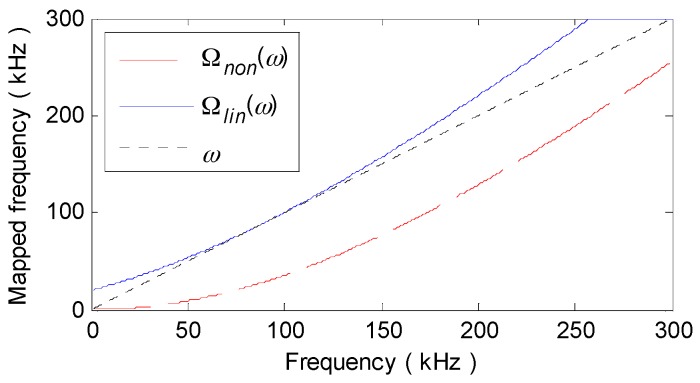
$\Omega_{lin}(\omega )$ and $\Omega_{non}(\omega )$.

To compensate $v_{26}(t)$, LDSC or NDSC is performed. The procedure is given in detail as follows:

(a) Determining parameters. With the parameters in Table 1, the wavenumber relation $K_{0}(\omega )$ of A_0_ mode in the aluminum plate is theoretically derived, as Figure 11 shows. Then, the group velocity at central frequency $c_{g}(90\;\mathrm{kHz})$ is computed as 1964.4 m/s and the linearized wavenumber relation $K_{lin}(\omega )$ or $K_{non}(\omega )$ is computed using Equation (6) or (9). The mapping sequence $\Omega_{lin}(\omega )$ or $\Omega_{non}(\omega )$ can be determined with Equation (14) or (16), as illustrated in Figure 12.

(b) $H_{26}\left[\Omega_{lin}(\omega )\right]$ or $H_{26}\left[\Omega_{non}(\omega )\right]$. Applying FFT to $h_{26}(t)$ and interpolating the resultant $H_{26}(\omega )$ with $\Omega_{lin}(\omega )$ or $\Omega_{non}(\omega )$, $H_{26}\left[\Omega_{lin}(\omega )\right]$ or $H_{26}\left[\Omega_{non}(\omega )\right]$ is obtained.

(c) $v_{26\_lin}(t)$ or $v_{26\_non}(t)$. Based on Equation (13) or (15), $v_{26\_lin}(t)$ or $v_{26\_non}(t)$ is finally computed.

As Figure 10b shows, either the A_0_ mode direct arrival or the reflections in $v_{26\_lin}(t)$ get well recompressed to the temporal sites relevant to their travelling times. Because of wavepacket recovery, their amplitudes are pronouncedly heightened without signal waveform deformation, as compared with $v_{26}(t)$ (seen in Figure 10a). Conversely, the residual S_0_ mode wavepacket in $v_{26\_lin}(t)$ (seen in the interval 50–100 μs in Figure 10b) is uncompensated but suffers more distortion and amplitude-attenuation. The reason for this is that LDSC is only performed with the A_0_ mode dispersion characteristic.

**Figure 13:**
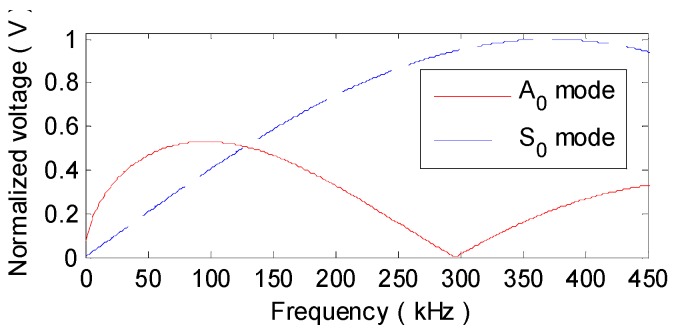
Theoretical amplitude-frequency response of A_0_ and S_0_ modes in the aluminum plate.

**Figure 14:**
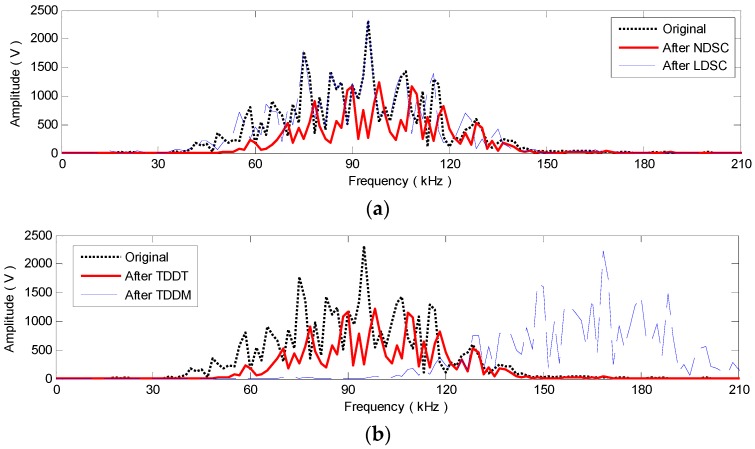
Amplitude spectra of original and processed sensor signals measured by $P_{2-6}$. (**a**) Amplitude spectra $A_{26}(r,\omega )$, $A_{26\_lin}(r,\omega )$, $A_{26\_non}(r,\omega )$ of $v_{26}(t)$, $v_{26\_lin}(t)$ and $v_{26\_non}(t)$; (**b**) Amplitude spectra $A_{26}(r,\omega )$, ${\tilde{A}}_{26}^{\prime }(r,\omega )$, $A_{26}^{\prime }(r,\omega )$ of $v_{26}(t)$, ${\tilde{v}}_{26}(r)$ and $v_{26}(r)$.

In $v_{26\_non}(t)$, as Figure 10c illustrates, though the dispersion effect on A_0_ mode waves are removed, the amplitudes of the recompressed wavepackets are even lower than those of the dispersive ones in $v_{26}(t)$. This is mainly attributed to the high degree of alteration in the amplitude spectrum $A_{26}(r,\omega )$ of $v_{26}(t)$ during NDSC. Figure 12 shows that, the mapped frequency point $\Omega_{non}(\omega )$ is far from the former one ω and especially, $\Omega_{non}(90\;\mathrm{kHz})$ at the central frequency 90 kHz is migrated to much lower 29 kHz. According to the A_0_ mode amplitude-frequency response computed with the theoretical formula in [16], as Figure 13 shows, $A_{26}(r,\omega )$ around 29 kHz is obviously weaker than that around 90 kHz. The amplitude spectrum $A_{26\_non}(r,\omega )$ of $v_{26\_non}(t)$ would dramatically decline with distinct variation tendency in contrast to $A_{26}(r,\omega )$, as Figure 14a illustrates. This can definitely decrease the amplitude of $v_{26\_non}(t)$.

By comparison, $\Omega_{lin}(\omega )$ cross the −3 dB bandwidth (68–112 kHz) of $v_{26}(t)$ deviates much less from ω and even overlaps with ω at $f_{c}$ = 90 kHz, as shown in Figure 12. Thus, the amplitude spectrum $A_{26\_lin}(r,\omega )$ of $v_{26\_lin}(t)$ is scarcely changed after LDSC and is almost the same as $A_{26}(r,\omega )$, as Figure 14a shows. Note that, the amplitude of S_0_ mode is so small in lower frequencies (seen in the interval 16.3–42.7 kHz in Figure 13) that the direct arrival of the mode nearly disappears in $v_{26\_non}(t)$ (seen in the interval 50–100 μs in Figure 10c). For the convenience of comparison, the envelopes of $v_{26}(t)$, $v_{26\_lin}(t)$ and $v_{26\_non}(t)$ are plotted together in Figure 10d. It can be obviously seen that, both the signal resolution and SNR are considerably improved by LDSC. Whereas, NDSC can only compress the A_0_ mode wavepacket without any signal energy reinforcement.

TDDM and TDDT are also executed on $v_{26}(t)$ to obtain the distance-domain signals ${\tilde{v}}_{26}(r)$ and $v_{26}(r)$, respectively. In ${\tilde{v}}_{26}(r)$, as Figure 10e shows, the A_0_ mode direct arrival and reflections are in compact wavepackets but with many more cycles. Evidently, ${\tilde{v}}_{26}(r)$ is disturbed by high frequencies. The signal waveform deformation of frequency shifting could decrease the compensation effect on ${\tilde{v}}_{26}(r)$. As Figure 10f shows, ignoring the discrepancy between independent variables, t and r, $v_{26}(r)$ is identical with $v_{26\_non}(t)$ (as illustrated in Figure 10c). After ${\tilde{v}}_{26}(r)$ and $v_{26}(r)$ are rescaling with $t=r/c_{g0}$, their amplitude spectra ${\tilde{A}}_{26}^{\prime }(r,\omega )$ and $A_{26}^{\prime }(r,\omega )$ can be also calculated. As Figure 14b shows, compared with $A_{26}(r,\omega )$, ${\tilde{A}}_{26}^{\prime }(r,\omega )$ is shifted toward a much higher frequency range and $A_{26}^{\prime }(r,\omega )$ has a much lower amplitude, which corresponds to the frequency shifting waveform deformation in ${\tilde{v}}_{26}(r)$ and the severe amplitude spectrum altering in $v_{26}(r)$, respectively.

#### 5.1.3. Compensation Effect on the Damage Scattered Signals

**Figure 15:**
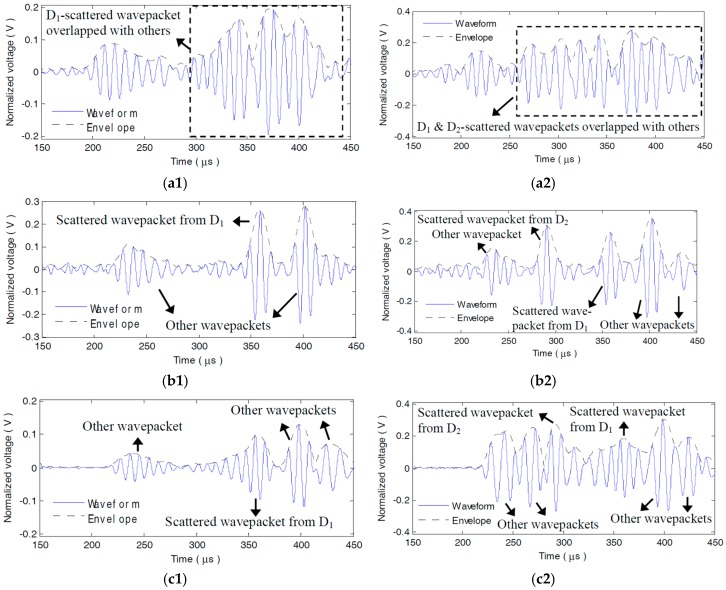
Original and processed damage scattered signals measured by $P_{5-8}$. (**a1**) Original scattered signal $s_{58}(t)$ from D_1_; (**a2**) Original scattered signal $s_{58}^{\prime }(t)$ from D_1_ and D_2_; (**b1**) LDSC-processed result $s_{58\_lin}(t)$ of $s_{58}(t)$ (**b2**) LDSC-processed result $s_{58\_lin}^{\prime }(t)$ of $s_{58}^{\prime }(t)$; (**c1**) NDSC-processed result $s_{58\_non}(t)$ of $s_{58}(t)$; (**c2**) NDSC-processed result $s_{58\_non}^{\prime }(t)$ of $s_{58}^{\prime }(t)$.

Subtracting the A_0_ mode sensor signals of the health plate from those of the damaged plate, the damage scattered signals can be obtained. Figure 15a1,a2 show the typical original dispersive scattered signals $s_{58}(t)$ and $s_{58}^{\prime }(t)$ from the single and dual adjacent damages measured by $P_{5-8}$, respectively. Due to dispersion, both the damage scattered wavepackets and the other wavepackets are elongated and seriously overlapped with each other in $s_{58}(t)$ and $s_{58}^{\prime }(t)$. It goes into an extraordinary challenge to interpret $s_{56}(t)$ or $s_{56}^{\prime }(t)$.

However, in the LDSC-processed result $s_{58\_lin}(t)$ of $s_{58}(t)$, as Figure 15b1 shows, the scattered wavepacket from D_1_ is recompressed to a 3-cycle sine burst and is distinctly separated from the right neighbouring wavepacket. While in $s_{56\_lin}^{\prime }(t)$, as Figure 15b2 illustrates, the two well-recovered wavepackets scattered by D_1_ and D_2_ can be readily distinguished with high enough amplitudes. Apparently, not only waveform reconversion but signal energy reinforcement is also accomplished for the dispersive scattered wavepackets *via* LDSC. The NDSC-processed results $s_{56\_non}(t)$ and $s_{56\_non}^{\prime }(t)$ are shown in Figure 15c1,c2, respectively. In $s_{56\_non}(t)$, the scattered wavepacket from D_1_ gets compensated, whereas the scattered wavepacket is decreased in its amplitude and grievously contaminated by other reflections or noises, as Figure 15c1 shows. Because of the amplitude attenuation, the two compensated damage scattered wavepackets are nearly submerged by other wavepackets and barely discriminated in $s_{56\_non}^{\prime }(t)$, especially for the one from D_1_, as Figure 15c2 shows. The reason for this is the severe alteration of the amplitude spectrum during NDSC.

#### 5.1.4. Damage Imaging Results

**Figure 16:**
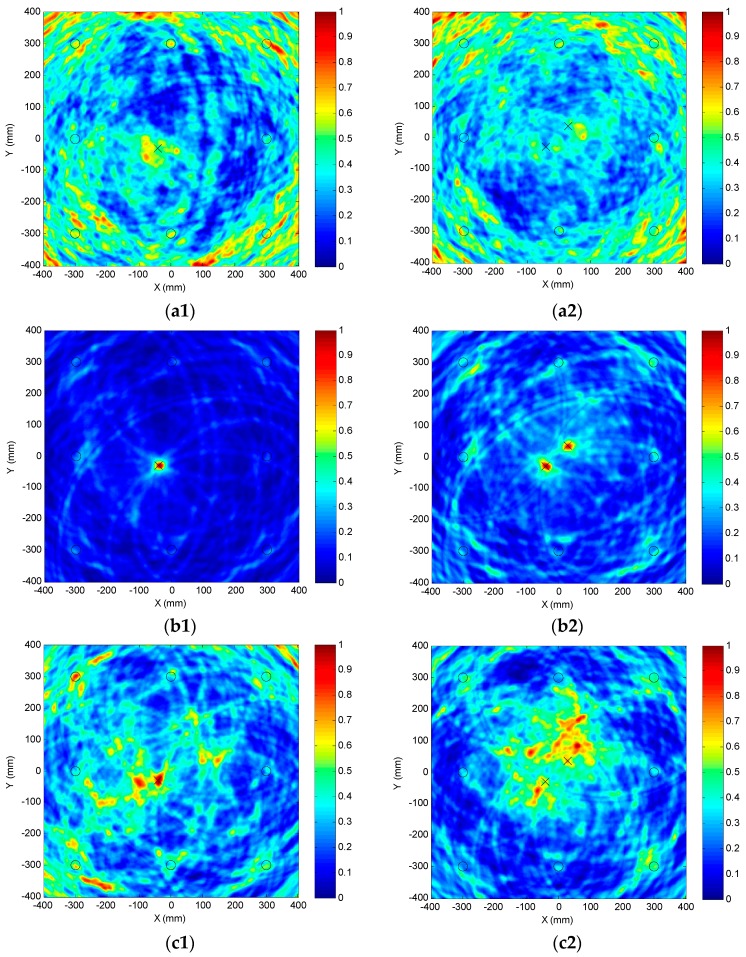
Different images of single and adjacent dual damages obtained by using traditional delay-and-sum, LDSC-based or NDSC-based imaging method. (**a1**) Original imaging result of D_1_; (**a2**) Original imaging result of D_1_ and D_2_; (**b1**) LDSC-based imaging result of D_1_; (**b2**) LDSC-based imaging result of D_1_ and D_2_; (**c1**) NDSC-based imaging result of D_1_; (**c2**) NDSC-based imaging result of D_1_ and D_2_.

Using the original, LDSC or NDSC-processed scattered signals acquired by all 28 PZT pairs of the sparse array, several images for the single or dual adjacent damages can be generated with Equations (27)–(29), as shown in Figure 16 where each symbol “X” denotes the actual damage position and the circulars denote the PZT wafers. With the dispersion effect on the scattered signals (seen in Figure 15a1,a2), no flaw spot can be clearly observed at each actual damage position in Figure 16a1,a2. The imaging for the single or dual damages obviously fails.

As the dispersion influence is efficiently removed in the LDSC-processed results (seen in Figure 15b1 and Figure 12b2), both the single damage and the dual neighboring ones can be visibly and accurately recognized as bright focalized spots in Figure 16b1 and Figure 13b2, showing the outstanding spatial resolution and SNR of the proposed LDSC-based damage imaging method. Due to the amplitude attenuation of the compensated scattered signals induced by the serious amplitude spectrum alteration (seen in Figure 15c1 and Figure 12c2), the flaw point is relatively blurred with a higher noise-level in the NDSC-based imaging result of D_1_, as illustrated in Figure 16c1. While in the image of D_1_ and D_2_, as Figure 16c2 shows, the SNR is too low to identify the two flaw spots.

### 5.2. Numerical Simulation of Quantitative Imaging

**Figure 17:**
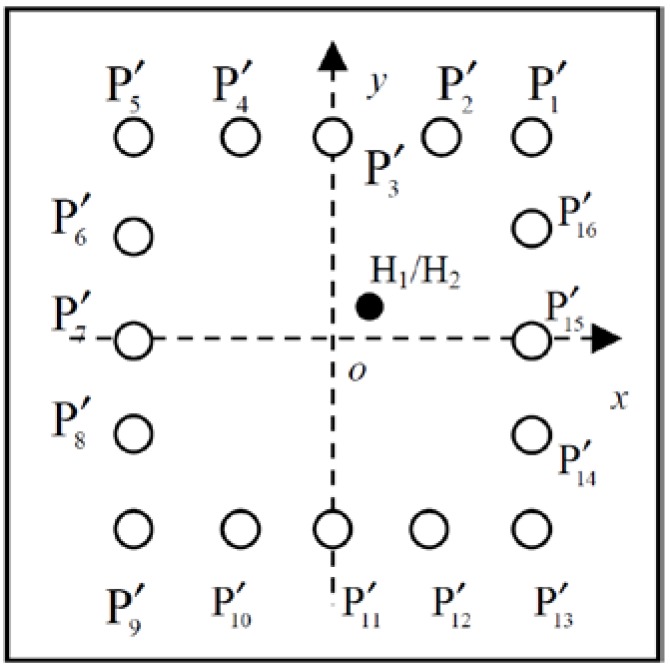
Distribution of the PZT array and holes in the aluminum plate.

With relatively more PZT wafers, the above high spatial resolution imaging methods can be attempted for quantitative imaging, in which besides locations, the other particular damage properties, e.g., severities or geometric dimensions, can be attained. In the numerical simulation, because of the excellent dispersion compensation capacity, LDSC is adopted for quantitatively imaging a rectangular hole H_1_ (50 mm × 60 mm) or circular hole H_2_ (40 mm in diameter) in a 1000 mm × 1000 mm × 1.5 mm aluminum plate with an array of 16 PZT wafers $P_{1}^{\prime }$~$P_{16}^{\prime }$, as shown in Figure 17. Table 3 gives the exact positions of the PZT wafers and holes. The simulation is conducted by the commercial finite element modeling (FEM) software ABAQUS^®^/EXPLICITE. The plate is modeled using the A4 node shell elements (S4) with the material parameters in Table 1. The step pulse excitation signal with the raising time of 0.25 μs is loaded as the opposing out-of-plane point-source and the deviation of the out-of-plane strain at each sensing point is calculated as the impulse response with the fixed time step of 0.5 μs. To extract the A_0_ mode dominated sensor signal from the impulse response of every PZT pair, the same 3-cycle sine burst excitation signal centered at 90 kHz is used.

**Figure 18:**
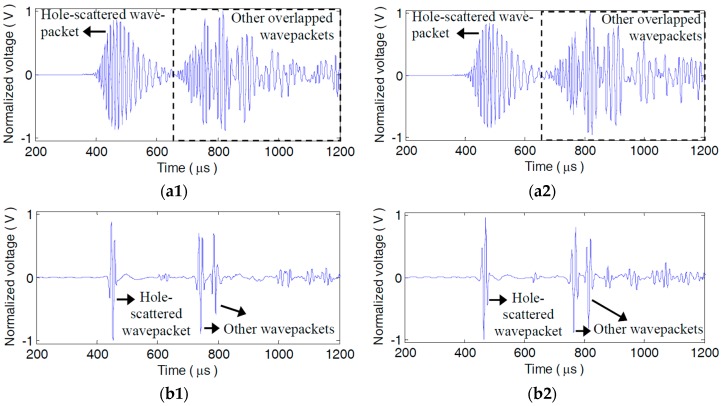
Original and LDSC-processed damage scattered signals from the rectangular or circular hole measured by $P_{1-7}^{\prime }$. (**a1**) Original scattered signal $s_{17}(t)$ from H_1_; (**a2**) Original scattered signal $s_{17}^{\prime }(t)$ from H_2_; (**b1**) LDSC-processed result $s_{17\_lin}(t)$ of $s_{17}(t)$; (**b2**) LDSC-processed result $s_{17\_lin}^{\prime }(t)$ of $s_{17}^{\prime }(t)$.

**Figure 19:**
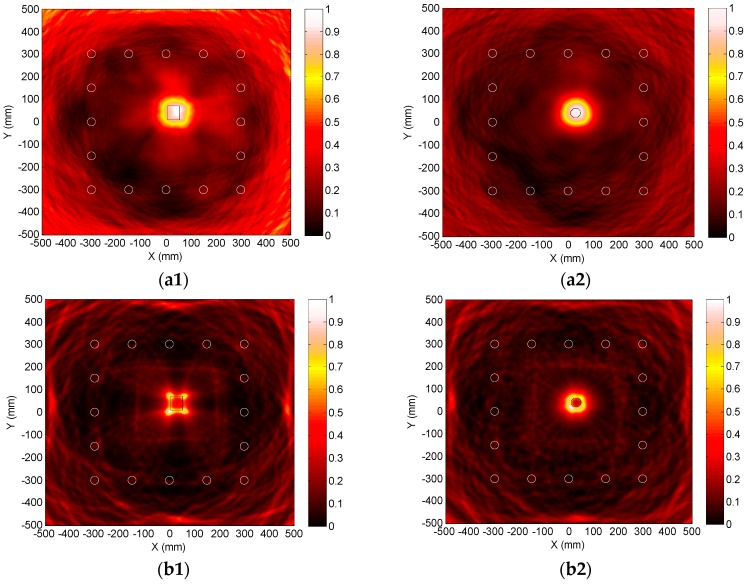
Different images of the rectangular hole H_1_ or circular one H_2_ respectively obtained by using traditional delay-and-sum or LDSC-based imaging method. (**a1**) Original imaging result of H_1_; (**a2**) Original imaging result of H_2_; (**b1**) LDSC-based imaging result of H_1_; (**b2**) LDSC-based imaging result of H_2_.

The dispersive scattered signals from H_1_ or H_2_ are compensated by LDSC with the interpolation sequence $\Omega_{lin}(\omega )$ in Figure 12. Figure 18a1,a2 illustrate the original scattered signals $s_{17}(t)$ and $s_{17}^{\prime }(t)$ from H_1_ or H_2_ measured by $P_{1-7}^{\prime }$. Their corresponding LDSC-processed results are respectively shown in Figure 18b1,b2, in which the impressive recompression for the hole-scattered and other wavepackets of A_0_ mode is observed. In the images computed with Equation (27) using all 120 original scattered signals from H_1_ or H_2_, as Figure 19a1,a2 show, the two holes are displayed as the expanded spots with obscure boundaries. Except for the gross sites, the more detailed flaw information cannot be further identified due to the poor imaging resolution. Using all the LDSC-processed scattered signals, H_1_ and H_2_ can be imaged with satisfactory spatial resolution based on Equation (28), as illustrated in Figure 19b1,b2, where the outer edges of the holes are also plotted. The flaw spots are in good coincidence with the hole locations, sizes and shapes, which can enable the following attractive quantitative identification for the hole damage. Note that, since the four corners of the rectangular hole are the most remarkable scattering sources for A_0_ mode Lamb waves, the scattered wavepackets are preferentially focalized on them to yield the four brightest points in Figure 19b1.

## 6. Conclusions

To overcome the nonlinear-dispersion problem in Lamb wave identification of aerospace structures, two signal construction methods, LDSC and NDSC are presented with the conception of nonlinear wavenumber linearization. Furthermore, the effects of the compensation process on the other crucial signal properties, especially the signal waveform and amplitude spectrum are particularly concerned. A comparison with TDDM and TDDT is also performed. The investigation results indicate that, with signal waveform suitably corrected and amplitude spectrum nearly unchanged, the dispersive signals can be preferably compensated by LDSC. Both the signal resolution and signal energy can be highly enhanced in the LDSC results. In either NDSC or TDDT, though signal waveform correction is realized, the amplitude spectrum is severely altered. This would result in the amplitude attenuation to the compensated signals and limit the application of NDSC or TDDT under low SNR circumstances. Without signal waveform correction, the recompressed wavepackets in TDDM-processed signals can be easily deformed with other frequency components, which could probably cause much inconvenience to the signal interpretation.

Hereafter, a LDSC or NDSC-based imaging method is further developed for high spatial resolution or quantitative damage imaging, respectively. Due to the amplitude attenuation in the ND-SC-processed damage scattered wavepackets, NDSC-based imaging for the actual adjacent dual flaws in the aluminum plate failed with serious noise, while the two flaws can be clearly displayed with high spatial resolution and SNR using the LDSC-based imaging method. Because of the outstanding dispersion compensation ability to keep other signal characteristics unchanged, satisfactory quantitative results of the rectangular or circular hole can be also attained by LDSC-based imaging with relatively more PZT wafers in the numerical simulation.

## Figures and Tables

**Table 1:** Material parameters of 2024 aluminum plate.

Density *ρ* (kg·cm^−3^)	Poisson’s Ratio *μ*	Yong’s Modulus *E* (Gpa)
2780	0.33	73.1

**Table 2:** The coordinates of PZTs and dual damages.

PZTs	(x, y)/(mm)	PZTs	(x, y)/(mm)
P_1_	(300, 300)	P_6_	(−300, 0)
P_2_	(−300, 300)	P_7_	(0, −300)
P_3_	(−300, −300)	P_8_	(300, 0)
P_4_	(300, −300)	D_1_	(−40, −30)
P_5_	(0, 300)	D_2_	(30, 35)

**Table 3:** The coordinates of PZTs and holes.

PZTs	(x, y)/(mm)	PZTs	(x, y)/(mm)
$P_{1}^{\prime }$	(300, 300)	$P_{10}^{\prime }$	(−150, −300)
$P_{2}^{\prime }$	(150, 300)	$P_{11}^{\prime }$	(0, −300)
$P_{3}^{\prime }$	(0, −300)	$P_{12}^{\prime }$	(150, −300)
$P_{4}^{\prime }$	(−150, −300)	$P_{13}^{\prime }$	(300, −300)
$P_{5}^{\prime }$	(−300, 300)	$P_{14}^{\prime }$	(300, −150)
$P_{6}^{\prime }$	(−300, 150)	$P_{15}^{\prime }$	(300, 0)
$P_{7}^{\prime }$	(−300, 0)	$P_{16}^{\prime }$	(300, 150)
$P_{8}^{\prime }$	(−300, −150)	H_1_	(30, 40)
$P_{9}^{\prime }$	(−300, −300)	H_2_	(30, 40)
